# Interventions After First Post-Transplant Cutaneous Squamous Cell Carcinoma: A Proposed Decision Framework

**DOI:** 10.3389/ti.2022.10880

**Published:** 2022-11-22

**Authors:** Matthew J. Bottomley, Paul R. Massey, Raj Thuraisingham, Alden Doyle, Swati Rao, Kristin P. Bibee, Jan Nico Bouwes Bavinck, Anokhi Jambusaria-Pahlajani, Catherine A. Harwood

**Affiliations:** ^1^ Chinese Academy of Medical Sciences Oxford Institute (CAMS-COI), Nuffield Department of Medicine, University of Oxford, Oxford, United Kingdom; ^2^ Oxford Transplant Unit, Oxford University Hospitals, NHS Foundation Trust, Oxford, United Kingdom; ^3^ Cheyenne Skin Clinic, Cheyenne, WY, United States; ^4^ Department of Renal Medicine and Transplantation, Barts Health NHS Trust, London, United Kingdom; ^5^ Department of Medicine, University of Virginia, Charlottesville, VA, United States; ^6^ Department of Dermatology, School of Medicine, John Hopkins University, Baltimore, MD, United States; ^7^ Department of Dermatology, Leiden University Medical Centre, Leiden, Netherlands; ^8^ Division of Dermatology, Department of Internal Medicine, Dell Medical School, The University of Texas at Austin, Austin, TX, United States; ^9^ Centre for Cell Biology and Cutaneous Research, Blizard Institute, Barts and The London School of Medicine and Dentistry, Queen Mary University of London, London, United Kingdom

**Keywords:** cancer, outcomes, transplant, skin cancer, management

## Abstract

Cutaneous squamous cell carcinoma (CSCC) is a major cause of morbidity and mortality after organ transplant. Many patients subsequently develop multiple CSCC following a first CSCC, and the risk of metastasis and death is significantly increased compared to the general population. Post-transplant CSCC represents a disease at the interface of dermatology and transplant medicine. Both systemic chemoprevention and modulation of immunosuppression are frequently employed in patients with multiple CSCC, yet there is little consensus on their use after first CSCC to reduce risk of subsequent tumors. While relatively few controlled trials have been undertaken, extrapolation of observational data suggests the most effective interventions may be at the time of first CSCC. We review the need for intervention after a first post-transplant CSCC and evidence for use of various approaches as secondary prevention, before discussing barriers preventing engagement with this approach and finally highlight areas for future research. Close collaboration between specialties to ensure prompt deployment of these interventions after a first CSCC may improve patient outcomes.

## A Clinical Case

A 60 year old white male presents for kidney transplant follow-up, 21 years after a deceased donor transplant. Despite an early cellular rejection episode, he has maintained excellent allograft function (baseline creatinine 107 μmol/L) without humoral sensitization on a dual regimen of cyclosporine and azathioprine. He has a history of photodamage but no history of skin cancer or solid-organ malignancy. He has recently had a 1 cm tender keratotic nodule excised from his shin, confirmed histologically as invasive cutaneous squamous cell carcinoma (CSCC). The patient asks whether anything can be done to decrease his risk of cancer recurrence without putting their allograft at undue risk.

## Introduction

Skin is the commonest site for post-transplant malignancy, with up to 200-fold increased incidence of keratinocyte carcinoma (KC) compared to immunocompetent populations (ICP) (1). CSCC accounts for 80% of KC in organ transplant recipients (OTR) (2). Half of OTR develop another CSCC within 3 years of their first (2–5). Metastatic risk from CSCC is doubled in OTR and those who develop multiple (>10) CSCC have up to 26% risk of metastasis (6, 7), with a 3 year median survival (8). CSCC represents a leading cause of cancer-related mortality for some OTR (2,8,9,10) and may be associated with increased risk of internal malignancies (11, 12), consistent with findings in ICP that are not fully explained by known cancer risk factors (13,14,15) and presumably relate to common susceptibility mechanisms. Treatment and surveillance for post-transplant CSCC creates significant economic burden for healthcare providers and patients (16). Interventions to reduce risk are desirable to improve OTR wellbeing, healthcare resource usage, and future cancer-related mortality.

**FIGURE 1:**
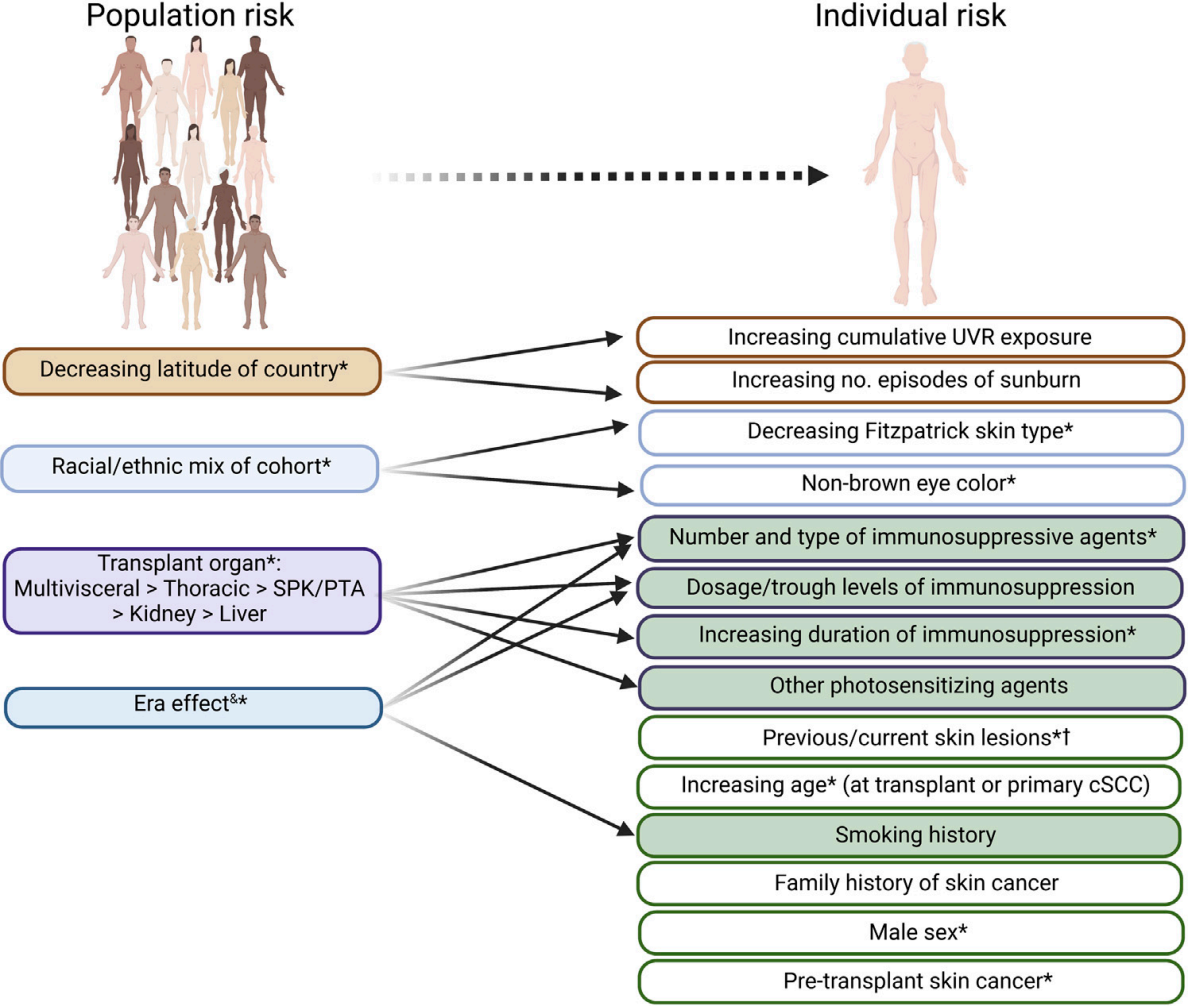
Clinical risk factors for (further) cSCC development, which may be useful in risk stratifying organ transplant recipients. Factors predictive of cSCC risk at a population level are indicated on the left. Factors relevant at an individual level (which are often interrelated—demonstrated by arrows) are shown on the right, with potentially modifiable factors at time of first cSCC shown in green. *indicates risk factors shown to be independently predictive of development of further KC or cSCC lesions in at least one study. cSCC, cutaneous squamous cell carcinoma. UVR; UV radiation; SPK, simultaneous kidney-pancreas; PTA, pancreas transplant alone. &time period during which cohort data is collected: generally more historical cohorts demonstrate greater cSCC risk. ^†^ including human papillomavirus (HPV)-related viral warts or dysplasia and UVR-related photodamage and pre-malignant lesions.

At a population level, cumulative incidence of CSCC amongst OTR is dependent upon several factors, the most important being immunosuppression intensity and geographic latitude (reflecting cumulative ultraviolet radiation (UVR) exposure) (2). 25% of white European OTR may ultimately develop CSCC, rising to 75% with significant UVR exposure (such as Australasia) (2). Pre-transplant CSCC is a major risk factor for post-transplant CSCC and consensus recommendations regarding the management of such patients have been published elsewhere (17). Individual risk factors are summarized in Figure 1. While used to guide cohort surveillance strategies (4, 18), prognostication using these factors [recently reviewed (19)], particularly for prediction of recurrence, lacks resolution to guide individual patient management.

We summarize staging of disease prevention for post-transplant skin cancer in Table 1 (20, 21). Primary and secondary prevention strategies for CSCC in OTR include patient education, photoprotection, clinical skin surveillance and topical and oral chemoprevention (22), though data in transplant cohorts are limited with recommendations extrapolated from relatively small studies (23–25), expert opinion (26, 27), or studies in ICP (28–30).

**TABLE 1:** Definition of stages of CSCC prevention used in this paper.

Prevention stage	Definition	Example(s) relevant to post-transplant CSCC
Primordial and primary	Prevent disease onset in susceptible individuals (i.e., with one or more risk factors)	Education regarding UV exposure, promoting use of photoprotection (such as sunscreen)
Secondary	Identify patients with early disease and prevent progression	Skin cancer screening, topical or systemic chemoprevention (including management of premalignant lesions) or modulation of immunosuppression in patient with first CSCC to prevent further CSCC.
Tertiary	Decrease morbidity and mortality of individuals with advanced disease	Surgery or radiotherapy to locally advanced lesions to prevent metastatic spread; immunotherapy for treatment of metastatic lesions
Quaternary	Protect individuals from medical interventions that may cause more harm than good	Avoiding sensitization and rejection resulting from immunosuppression modulation

Staging of disease prevention differs in post-transplant skin cancer compared to other diseases, where progression does not solely represent growth and metastasis of a single malignancy, but also the development of further asynchronous primary lesions. Summarized from references (20, 21, 31).

Uncertainty about optimal timing of these interventions led to formulation of expert consensus-based recommendations for management, including a recent international Delphi panel of transplant dermatologists (26). While consensus was reached regarding topical and systemic agents in primary and secondary prevention of CSCC, consensus was not reached for optimal interventions after a first low-risk CSCC (LRCSCC; defined in this study, and this paper, as Brigham and Women’s Hospital Stage T1 or T2a, or American Joint Committee on Cancer T1 or T2). Retrospective data suggest there is similar equipoise about optimal timing and nature of immunosuppressive regimen modification amongst transplant practitioners, particularly after first CSCC (3).

In the absence of definitive evidence, we provide an overview of potential interventions for secondary CSCC prevention after the first CSCC and suggest this timepoint as an optimal opportunity to consider initiation of such measures. We consider dermatology, transplant medicine and patient perspectives relevant to decision making and consider the current barriers to adoption of this practice. Finally, we propose a decision framework to guide management of after a first post-transplant CSCC.

## Dermatological Strategies

There is scant evidence to guide transplant dermatologists in predicting CSCC risk and employing secondary prevention measures in OTRs after their first LRCSCC. OTR with a history of CSCC should be counselled on skin self-examination and photoprotection and undergoing regular skin cancer surveillance (4, 18), though screening interval recommendations are not consistent across international guidelines. There is randomized controlled trial (RCT) evidence that regular use of sunscreen reduces the risk of first CSCC in ICP, but data for benefit in OTR are limited to case-control studies (32).

Actinic keratoses (AK) are clinically apparent hyperkeratotic papules and plaques representing epidermal dysplasia arising on sun-damaged skin; a small proportion proceed to invasive CSCC (0.01%–0.65% in ICP) (33). CSCC *in situ* (CSCCIS, Bowen disease) represents full-thickness epidermal dysplasia with a higher rate of transformation to CSCC (3%–5% in ICP) (34). AK and CSCCIS may become confluent in areas of ‘field cancerization’, with subclinical disease present in contiguous clinically normal photo-exposed skin. Management of premalignancy is an essential component of secondary prevention. Destructive therapies such as cryotherapy or surgical curettage and cautery tend to be favored for discrete lesions (24). In confluent areas of AK, topical “field directed” treatments are added (35). 5% 5-fluorouracil (5-FU) cream has demonstrated superiority in blinded trials over alternatives in ICP and has also been demonstrated to prevent CSCC (22, 35), with evidence of superiority in OTR limited but growing (29, 36, 37).

Dermatologists may consider oral chemoprevention for patients at high risk of subsequent CSCC, with options including oral retinoids (acitretin) or nicotinamide (26). Acitretin is effective with up to 42% reduction in rates of CSCC in kidney transplant recipients in RCTs (23, 25). However, reported rates of discontinuation due to side effects range from 19%–39% in RCTs of OTR, most commonly due to xerosis and alopecia (23, 25). “Rebound” CSCC formation 3–4 months after drug cessation is frequent, meaning acitretin should be regarded as a long-term strategy (38). These factors may account for part of the documented reluctance of dermatologists to start acitretin after a first CSCC, typically waiting until multiple/high-risk CSCC formation is evident (26). In Australian ICP with a history of multiple KC, oral nicotinamide (active vitamin B3) 500 mg twice daily was well tolerated and resulted in a 30% reduction in CSCC compared to placebo over 12 months, but also showed rebound effects upon discontinuation (24). Nicotinamide has been studied in two insufficiently powered RCTs in kidney transplant recipients (39), but concerns regarding lack of positive data has limited its broader use by dermatologists in OTR (26). Results from a larger Australian RCT are forthcoming. Neither nicotinamide nor acitretin have been associated with significant changes in kidney allograft function or risk of allosensitization.

## Modification of Immunosuppression

There are two immunosuppression-based secondary prevention strategies that may reduce risk of subsequent CSCC after a first CSCC: change of immunosuppressive agent or reduction in immunosuppressive intensity.

### Change of Agent

#### Switch to Newer Agents

The direct carcinogenicity of various immunosuppressive agents is well established, particularly with those used prior to the mid-2000s. Azathioprine promotes UVA absorption by DNA, leading to UVA photosensitivity, mutagenicity and a unique mutational signature within CSCC (40, 41). Whilst azathioprine use is largely historical, it is still used in cases of mycophenolate intolerance and in recipients planning pregnancy: furthermore, Furthermore, the lag effect of CSCC development after transplant means many OTR who develop CSCC are still on this agent. Previous studies suggest up to 10% of Australian and US kidney transplant recipients, and up to 69% of Spanish heart transplant recipients, are receiving azathioprine (42). Mycophenolate does not promote UVA sensitivity, though may inhibit DNA repair mechanisms (43). Cyclosporine, but not tacrolimus, impairs UVR-induced DNA damage repair and apoptotic mechanisms and promotes tumor growth in pre-clinical models (41, 44). A large retrospective analysis of OTR found increased skin cancer risk with both cyclosporine and azathioprine compared to tacrolimus and mycophenolate, respectively (45). More recent regimens of tacrolimus and mycophenolate may be associated with a significant reduction in skin cancer risk compared to historical regimens and transition from azathioprine to mycophenolate appears to reduce first CSCC risk (45, 46). A major limitation to evidence for efficacy of this approach for secondary prevention is that the previous studies have been observational only. Belatacept may be an alternative or adjunct to calcineurin inhibitors (CNI) in certain kidney transplant recipients. The impact of belatacept on skin cancer is still emerging with a small single-center study showing lower risk of additional skin cancers after conversion from CNI to belatacept maintenance (47).

#### Switch to mTOR Inhibitor

Mammalian target of rapamycin inhibitors (mTORi) are associated with anti-malignant effects through multiple pathways *in vitro* (41). Several small studies alongside two large multicenter randomized trials assessed the effect of switching from CNI to sirolimus for CSCC secondary prevention in kidney transplant recipients (48, 49). A 25%–40% reduction in further CSCC risk over 2-year was seen in those converted to sirolimus, though only one study achieved significance across the cohort, and this was seen only after the first but not subsequent CSCC (48). A single episode of borderline rejection was seen across both studies and 5-year follow-up suggested similar patient and graft survival, arguing immunosuppression transition is safe (50). However, sirolimus was generally poorly tolerated with discontinuation and crossover in around a third of recipients due to adverse effects and a CSCC rebound effect was observed. Adverse effects include significant proteinuria, pneumonitis, oedema, impaired wound healing, teratogenicity and hyperlipidaemia. A meta-analysis of 21 trials found mTORi therapy was associated with a significant 60% reduction in KC risk, but also an increased risk of mortality due to infection and cardiovascular disease, though this may be partly due to higher intensity mTORi regimens used in earlier studies (51). For these reasons, sirolimus has not become a mainstay of therapy for CSCC primary or secondary prevention. Recent data have suggested that an alternative mTORi, everolimus, may demonstrate comparable transplant outcomes in low and moderate-risk patients when used alongside low-dose calcineurin inhibition compared to standard immunosuppression (52), and this may reignite interest in the use of mTORi as an immunosuppressant. Analysis of long-term outcomes from earlier studies suggest everolimus is broadly similar to sirolimus in efficacy in reducing KC burden, though tolerability remains a concern (53, 54).

### Reduction in Immunosuppression Intensity

**FIGURE 2:**
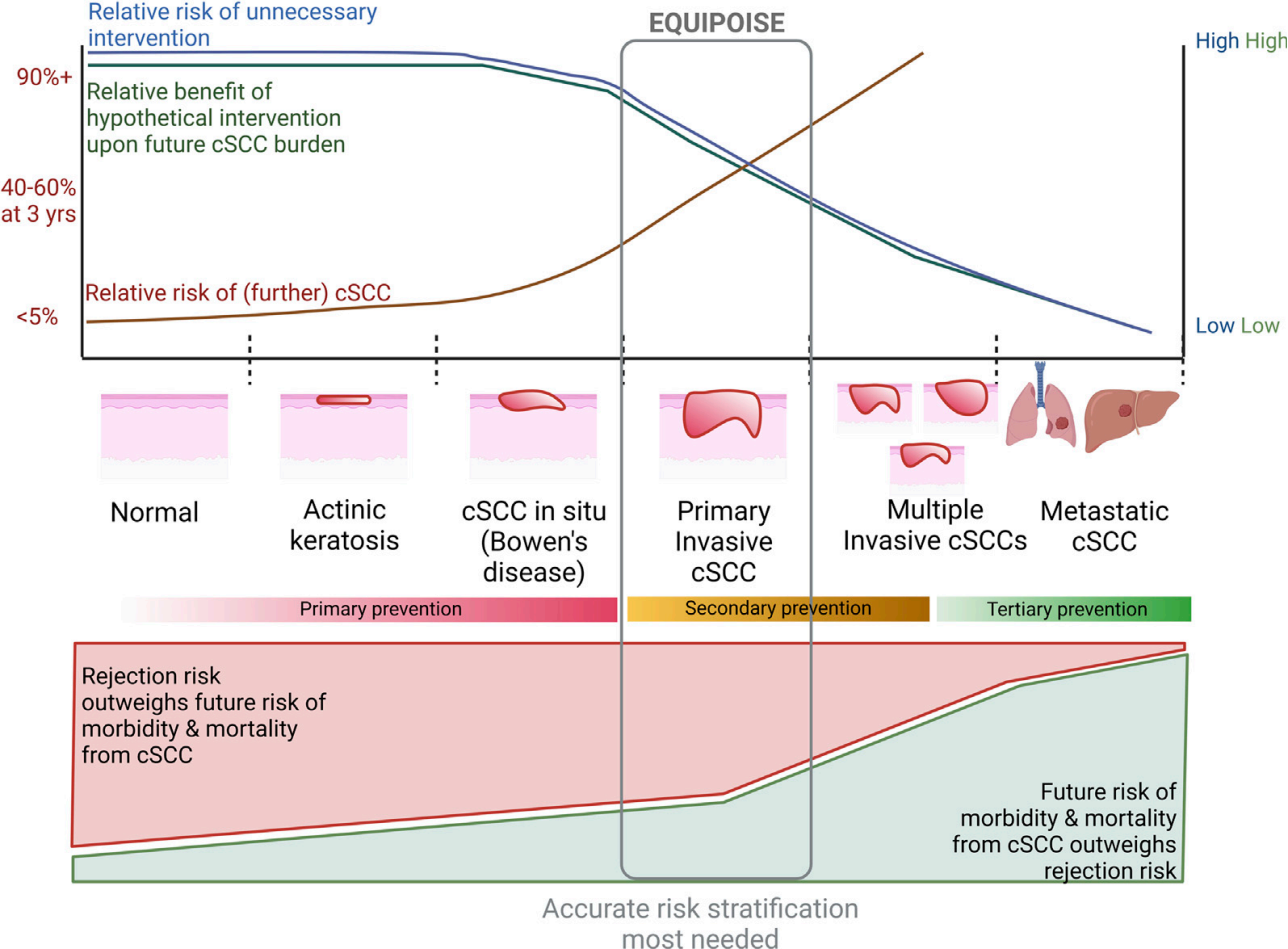
Considerations in utilizing a hypothetical intervention for reduction of cSCC risk at various stages of disease. Top graph indicates relative risk of future cSCC at a patient level (approximate figures given on Y-axis) and unnecessary intervention, as well as relative benefit upon future malignancy risk from intervention at each stage of squamous carcinogenesis. This graph represents an extrapolation of trial and observational data. Bottom graph represents relative risk of morbidity and mortality from future cSCC and rejection with immunosuppression modulation. We postulate equipoise is greatest at time of first cSCC for most OTR, by which time the risk of further cSCC is high enough that more accurate methods of risk stratification are needed to delineate whether rejection upon immunosuppression modulation or future malignancy are more likely.

**FIGURE 3:**
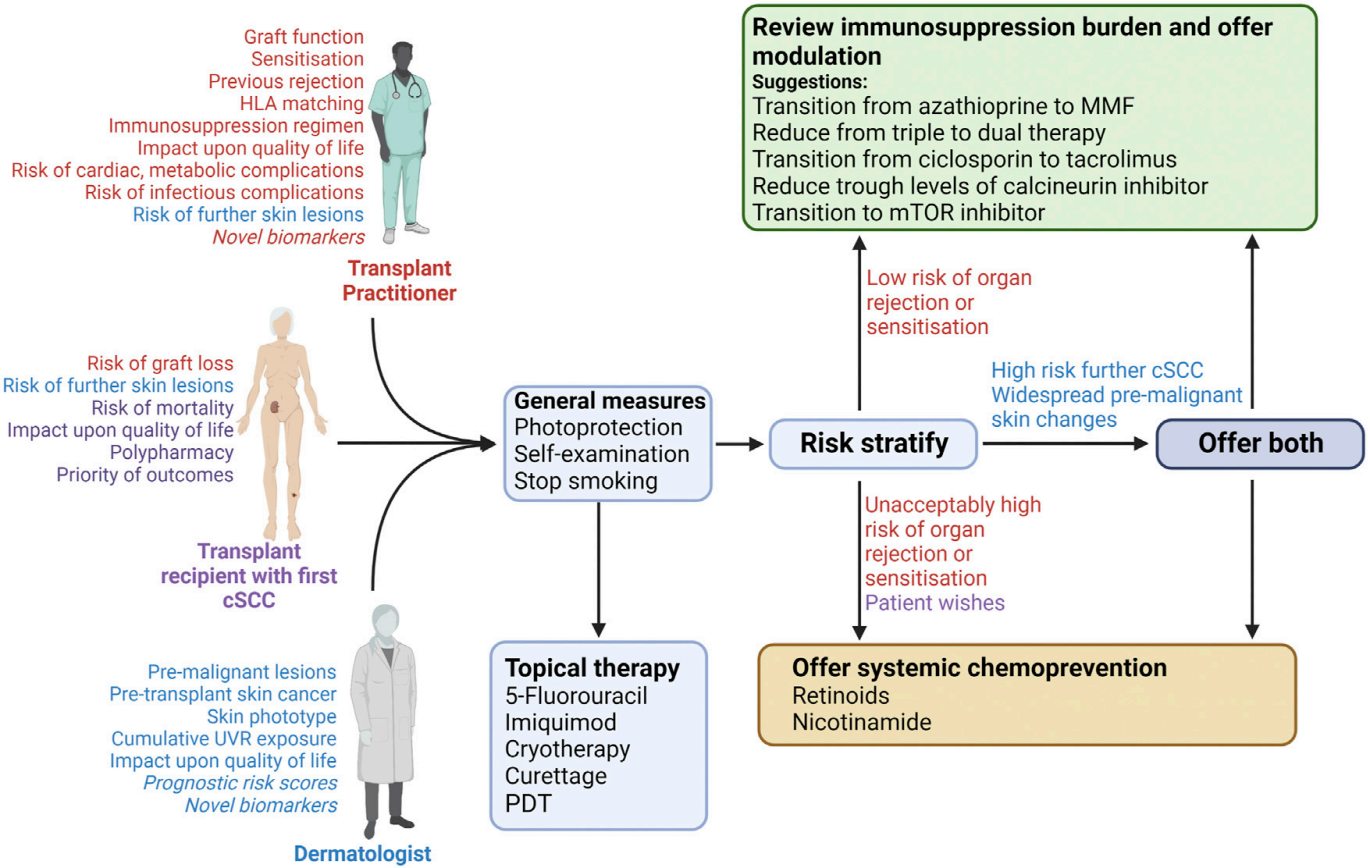
Approach to risk stratification and interventions after primary cSCC in an organ transplant recipient. Free-text indicates the important considerations by each member of the discussion (indicated by colour coding: red will be mostly guided by transplant practitioner, purple by the patient, and blue by the dermatologist. Those considerations in italics are not in widespread use but may become relevant in the future). Discussion between the recipient, dermatologist and transplant practitioner should lead to lifestyles changes and the treating dermatologist should offer topical therapy for other lesions, irrespective of perceived cSCC risk. The respective clinicians should subsequently consider cSCC risk alongside perceived risk of allograft rejection/sensitisation. The relative risks of these will guide the offer of immunosuppression modulation and/or systemic chemoprevention. Final discussion between the dermatologist and transplant practitioner will guide on the final interventions offered to the patient. cSCC; cutaneous squamous cell carcinoma; CNI, calcineurin inhibitor; PDT, photodynamic therapy; UVR, UV radiation.

When considering reduction in immunosuppression intensity, the transplant practitioner may consider factors including graft function, pre-existing sensitization and history of rejection episodes, and perceived balance between rejection and future malignancy risk (Figures 2, 3). A major limitation is the lack of methods to determine ‘optimal’ immunosuppression intensity at an individual level. Novel markers to stratify rejection risk are currently being developed, including circulating/urinary transcriptomics, HLA eplet mismatch profiling and donor-derived cell-free DNA [recently reviewed in (55)], but are not in widespread use and require validation regarding utility in guiding immunosuppression reduction.

Immunosuppression intensity is often related to clinical circumstances, including organ transplant type, and is correlated with first CSCC risk: for example, recipients on dual immunosuppression or with lower CNI trough levels exhibit reduced skin cancer risk compared to counterparts on triple immunosuppression or with greater trough levels (56, 57). Immunosuppression reduction or cessation (following graft failure) is associated with reduced risk and improved outcomes for virus-associated post-transplant malignancy such as lymphoma and Kaposi sarcoma (58), presumably by allowing greater immune control of cancer-associated viruses (59). However, data to support this approach for secondary prevention of CSCC is limited to retrospective cohort analyses, usually for advanced disease (3, 56). Immunosuppression modulation could synergize with chemopreventative approaches by permitting enhanced immune responses, but a combined approach has not been explored in either observational or trial settings.

## Timing of Interventions

In theory, the earlier the interventions are undertaken, the slower the accumulation of mutations developing, reducing risk of CSCC development.

A landmark trial showed reduction in CNI intensity at 1-year post-transplant was associated with reduced rates of malignancy over the following 5 years, of which two-thirds were skin cancer (57). While associated with an increased rate of acute rejection, this did not appear to compromise graft survival, possibly due to a relatively low event rate, and relatively high trough concentrations (by current standards) of cyclosporine in the intervention arm. Rates of *de novo* donor-specific antibodies, a marker of allosensitization that reflects under-immunosuppression, or of further CSCC were not assessed. Intensity of cyclosporine therapy in the intervention (low dose) arm was roughly equivalent to that currently used and so whether even further reduction would benefit CSCC risk without compromising graft outcomes is uncertain as is the benefit of reduced doses of tacrolimus.

The most effective intervention timepoint may be before the first CSCC and when premalignant lesions are diagnosed. However, the risk of destabilizing graft function or introducing side-effects with immunosuppression modulation is likely greater than the potential benefit and in most cases quaternary prevention is more relevant (Table 1; Figure 2). Specifically, refractory cellular rejection through excessive immunosuppression reduction may require use of lymphocyte depleting monoclonal antibodies; the use of these at time of transplant as induction therapy is associated with increased risk of subsequent malignancy and it is reasonable to assume the same untoward shift in risk when used as rescue therapy in rejection, though increased CSCC risk has not been demonstrated directly (60).

In contrast, OTRs with a first CSCC are at high risk of further CSCC, representing the optimal time to modulate immunosuppression in most cases. This benefit may extend beyond the skin by impacting common underlying mechanisms responsible for both CSCC and solid organ malignancy (11–15). However, the risks of immunosuppression modulation based upon skin malignancy should be weighed against the ‘number needed to treat’ to prevent future skin and internal malignancy (Figure 2).

As indicated above, RCTs investigating CNI to sirolimus transition demonstrated that OTR with a single CSCC versus multiple CSCC at randomization gained the greatest benefit from a switch to sirolimus, with a striking 90% reduction in CSCC risk over the following 2 years (48–50).

These data indirectly suggest that immunosuppression modulation could be the most effective secondary prevention strategy, if implemented in a timely fashion. We suggest that after a first SCC, OTRs should be considered for transition off older agents, particularly azathioprine. Reduction of CNI target levels may also be appropriate. Sirolimus may be an option for those perceived to be at high risk of multiple subsequent CSCC, but tolerance is a major barrier.

## Considerations of the Patient

While the patient will rely on the dermatologist and transplant physician to counsel regarding relative risks, it is important to consider the patient’s perspective.

The median time to first CSCC is typically many years after transplant, unless they have a pre-transplant history of CSCC (2); therefore, any intervention will generally be undertaken in the context of relatively stable graft function. Many OTR harbor an ongoing fear of rejection (61). Studies have found differences in prioritization of graft survival above other outcomes, including cancer and death (61–63), indicating outcomes of importance vary at a patient level. Many of the prevention tools available from a dermatology perspective do not incur risk for rejection but do warrant counselling on side effects and rebound CSCC upon drug cessation. Changes in immunosuppression may pose a rejection risk. While treatment of acute cellular rejection has good outcomes if detected rapidly, under-immunosuppression leading to humoral allosensitization is associated with significantly poorer graft survival and there is no consensus regarding effective treatment (64). Transplant recipients may be reluctant to change immunosuppression without individualized counselling balancing risk and benefits of this approach (61). Such counselling is difficult at present without more accurate CSCC risk stratification tools. Where immunosuppression modulation could be helpful, patients should be counselled regarding the uncertainty of individually predicting future CSCC risk, whilst emphasizing that a first CSCC is frequently associated with development of further lesions. Immunosuppression modulation at this timepoint may represent the optimal time to intervene and may also reduce the risk for other cancers, albeit with limited data to support this. Immunosuppression adjustment should be cautious and stepwise with close monitoring for graft function and sensitization.

## How do We Overcome Equipoise?

Two barriers contribute to clinical equipoise regarding secondary prevention: the need for risk stratification and evidence to guide sequencing of preventative strategies.

Perhaps most important is the need for accurate risk stratification, both for further CSCC and rejection. Cohort studies demonstrate that the majority of OTRs with CSCC will form multiple tumors over a 10-year period (4, 6, 7). Risk stratification is critical for formulating secondary prevention interventions, especially as these must be balanced against allograft function. One approach would be to develop more accurate clinical prediction tools based on algorithms to prioritize skin cancer screening and interval surveillance following transplantation (4, 18). Increased intensity of dermatology follow-up in highest-risk cohorts would allow for earlier lesion detection but also an opportunity to initiate intervention with effective field therapies and discussion of chemoprevention agents.

Development of novel biomarkers to facilitate more accurate risk stratification after first CSCC as a complementary approach would serve two purposes: identification of those most likely to benefit from interventions and enrichment of trials with those at greatest risk. A full review of potential biomarkers is beyond the scope of this article. However, circulating immunological markers have been of interest as neoantigens that may drive immunological responses are common (especially in premalignancy) due to the high mutational burden in CSCC and the possible association with HPV (65). Other markers, including polygenic risk scores (66, 67), polymorphisms identified through genome-wide association studies (67–71), circulating (and tumoral) microRNA (72) and tumoral gene expression (73, 74) have been investigated for prognostic value in either OTR or ICP. Only a subset have been validated externally and/or for stratification of further CSCC risk (66, 67, 75, 76, 77). Synchronous stratification for rejection risk would reassure both practitioners and patients regarding immunosuppression reduction.

A second barrier is the lack of clarity regarding relative effectiveness of interventions to reduce secondary CSCC risk and how these should be sequenced. Several dermatological approaches are available to mitigate risk of second CSCC, but studies are limited. For immunosuppression, a single center retrospective study identified 24 different immunosuppression minimization strategies undertaken after first CSCC in kidney and heart transplant recipients (3). Since the sirolimus studies in the 2000s, interventional trials of immunosuppression modification for secondary CSCC risk reduction have been absent. What trial designs might address this? The “Randomised Evaluation of COVID-19 therapy (RECOVERY)’ trial offers some inspiration: utilizing a simple design, central randomization with broad inclusion criteria and an adaptive trial platform design facilitated rapid, multi-center enrolment with a hard (mortality) endpoint to compare a series of possible treatments with established best care (78). A similar approach could facilitate a coordinated platform study of dermatological interventions after a first CSCC alongside immunosuppression modulation with the endpoint of subsequent CSCC (or locoregional recurrence/distant metastasis) development. The majority of subsequent CSCC development and poor outcomes are within the first 3 years of the first (2, 4), allowing for a medium-term follow-up period. The historical variety of immunosuppressive regimens have reduced over the last 20 years, coalescing around the use of tacrolimus, mycophenolic acid and/or corticosteroids, reducing the number of combinations to consider, though novel agents such as belatacept, proteosome inhibitors, IL-6 blockade and others may lead to future diversification of regimens.

## Conclusion

In summary, while CSCC management is often considered complete after excision, we propose that the first CSCC diagnosis should be regarded as a “red flag” heralding an increased risk of further skin cancers and possibly internal malignancies. It therefore represents a key opportunity to proactively consider secondary preventive strategies, although as optimal preventative interventions and their sequencing remain unclear, further research is needed.

As summarized in Figure 3, based on existing evidence, we recommend that dermatologists should routinely communicate with the transplant team after diagnosis of a first post-transplant CSCC. This event should spark a discussion regarding risk of further lesions, with review of immunosuppression burden and use of chemopreventative therapies. This dialogue between dermatologists, transplant practitioners and patients should be viewed as part of an ongoing shared decision-making process, with the ultimate aim of reducing skin cancer risk, ensuring optimal allograft function and ultimately improving survival and quality of life.

## Data Availability

The original contributions presented in the study are included in the article/supplementary material, further inquiries can be directed to the corresponding author.

## References

[1] Garrett GL Blanc PD Boscardin J Lloyd AA Ahmed RL Anthony T Incidence of and Risk Factors for Skin Cancer in Organ Transplant Recipients in the United States. JAMA Dermatol (2017) 153(3):296–303. 10.1001/jamadermatol.2016.4920 28097368

[2] Madeleine MM Patel NS Plasmeijer EI Engels EA Bouwes Bavinck JN Toland AE Epidemiology of Keratinocyte Carcinomas after Organ Transplantation. Br J Dermatol (2017) 177(5):1208–16. 10.1111/bjd.15931 28994104

[3] Euvrard S Kanitakis J Decullier E Butnaru AC Lefrancois N Boissonnat P Subsequent Skin Cancers in Kidney and Heart Transplant Recipients after the First Squamous Cell Carcinoma. Transplantation (2006) 81(8):1093–100. 10.1097/01.tp.0000209921.60305.d9 16641592

[4] Harwood CA Mesher D McGregor JM Mitchell L Leedham-Green M Raftery M A Surveillance Model for Skin Cancer in Organ Transplant Recipients: a 22-year Prospective Study in an Ethnically Diverse Population. Am J Transpl (2013) 13(1):119–29. 10.1111/j.1600-6143.2012.04292.x 23072567

[5] Wehner MR Niu J Wheless L Baker LX Cohen OG Margolis DJ Risks of Multiple Skin Cancers in Organ Transplant Recipients: A Cohort Study in 2 Administrative Data Sets. JAMA Dermatol (2021) 157(12):1447–55. 10.1001/jamadermatol.2021.4148 34668933 PMC8529524

[6] Levine DE Karia PS Schmults CD. Outcomes of Patients with Multiple Cutaneous Squamous Cell Carcinomas: A 10-Year Single-Institution Cohort Study. JAMA Dermatol (2015) 151(11):1220–5. 10.1001/jamadermatol.2015.1702 26177278

[7] Gonzalez JL Reddy ND Cunningham K Silverman R Madan E Nguyen BM. Multiple Cutaneous Squamous Cell Carcinoma in Immunosuppressed vs Immunocompetent Patients. JAMA Dermatol (2019) 155(5):625–7. 10.1001/jamadermatol.2018.5595 30865240 PMC6506878

[8] Martinez JC Otley CC Stasko T Euvrard S Brown C Schanbacher CF Defining the Clinical Course of Metastatic Skin Cancer in Organ Transplant Recipients: a Multicenter Collaborative Study. Arch Dermatol (2003) 139(3):301–6. 10.1001/archderm.139.3.301 12622621

[9] Venables ZC Autier P Nijsten T Wong KF Langan SM Rous B Nationwide Incidence of Metastatic Cutaneous Squamous Cell Carcinoma in England. JAMA Dermatol (2019) 155(3):298–306. 10.1001/jamadermatol.2018.4219 30484823 PMC6521686

[10] Garrett GL Lowenstein SE Singer JP He SY Arron ST. Trends of Skin Cancer Mortality after Transplantation in the United States: 1987 to 2013. J Am Acad Dermatol (2016) 75(1):106–12. 10.1016/j.jaad.2016.02.1155 27067869

[11] Zamoiski RD Yanik E Gibson TM Cahoon EK Madeleine MM Lynch CF Risk of Second Malignancies in Solid Organ Transplant Recipients Who Develop Keratinocyte Cancers. Cancer Res (2017) 77(15):4196–203. 10.1158/0008-5472.CAN-16-3291 28615224 PMC5540772

[12] Wisgerhof HC Wolterbeek R De Fijter JW Willemze R Bouwes Bavinck JN. Kidney Transplant Recipients with Cutaneous Squamous Cell Carcinoma Have an Increased Risk of Internal Malignancy. J Invest Dermatol (2012) 132(9):2176–83. 10.1038/jid.2012.132 22534875

[13] Zheng G Sundquist K Sundquist J Forsti A Hemminki A Hemminki K. Incidence Differences between First Primary Cancers and Second Primary Cancers Following Skin Squamous Cell Carcinoma as Etiological Clues. Clin Epidemiol (2020) 12:857–64. 10.2147/CLEP.S256662 32821171 PMC7417931

[14] Wheless L Black J Alberg AJ. Nonmelanoma Skin Cancer and the Risk of Second Primary Cancers: a Systematic Review. Cancer Epidemiol Biomarkers Prev (2010) 19(7):1686–95. 10.1158/1055-9965.EPI-10-0243 20570907 PMC2901413

[15] Rees JR Zens MS Gui J Celaya MO Riddle BL Karagas MR. Non Melanoma Skin Cancer and Subsequent Cancer Risk. PLoS ONE (2014) 9(6):e99674. 10.1371/journal.pone.0099674 24937304 PMC4061037

[16] Gordon LG Rodriguez‐Acevedo AJ Papier K Khosrotehrani K Isbel N Campbell S The Effects of a Multidisciplinary High‐throughput Skin Clinic on Healthcare Costs of Organ Transplant Recipients. J Eur Acad Dermatol Venereol (2019) 33(7):1290–6. 10.1111/jdv.15458 30706970

[17] Zwald F Leitenberger J Zeitouni N Soon S Brewer J Arron S Recommendations for Solid Organ Transplantation for Transplant Candidates with a Pretransplant Diagnosis of Cutaneous Squamous Cell Carcinoma, Merkel Cell Carcinoma and Melanoma: A Consensus Opinion from the International Transplant Skin Cancer Collaborative (ITSCC). Am J Transpl (2016) 16(2):407–13. 10.1111/ajt.13593 26820755

[18] Jambusaria-Pahlajani A Crow LD Lowenstein S Garrett GL Melcher ML Chan AW Predicting Skin Cancer in Organ Transplant Recipients: Development of the SUNTRAC Screening Tool Using Data from a Multicenter Cohort Study. Transpl Int (2019) 32(12):1259–67. 10.1111/tri.13493 31423648

[19] Lowenstein SE Garrett G Toland AE Jambusaria‐Pahlajani A Asgari MM Green A Risk Prediction Tools for Keratinocyte Carcinoma after Solid Organ Transplantation: a Review of the Literature. Br J Dermatol (2017) 177(5):1202–7. 10.1111/bjd.15889 28952162

[20] Perez M Abisaad JA Rojas KD Marchetti MA Jaimes N. Skin Cancer: Primary, Secondary, and Tertiary Prevention. Part I. J Am Acad Dermatol (2022) 87(2):255–68. 10.1016/j.jaad.2021.12.066 35176397

[21] Rojas KD Perez ME Marchetti MA Nichols AJ Penedo FJ Jaimes N. Skin Cancer: Primary, Secondary, and Tertiary Prevention. Part II. J Am Acad Dermatol (2022) 87(2):271–88. 10.1016/j.jaad.2022.01.053 35176398

[22] Chung EYM Palmer SC Strippoli GFM. Interventions to Prevent Nonmelanoma Skin Cancers in Recipients of a Solid Organ Transplant: Systematic Review of Randomized Controlled Trials. Transplantation (2019) 103(6):1206–15. 10.1097/TP.0000000000002641 31246934

[23] Bavinck JN Tieben LM Van der Woude FJ Tegzess AM Hermans J ter Schegget J Prevention of Skin Cancer and Reduction of Keratotic Skin Lesions during Acitretin Therapy in Renal Transplant Recipients: a Double-Blind, Placebo-Controlled Study. J Clin Oncol (1995) 13(8):1933–8. 10.1200/JCO.1995.13.8.1933 7636533

[24] Chen AC Martin AJ Choy B Fernandez-Penas P Dalziell RA McKenzie CA A Phase 3 Randomized Trial of Nicotinamide for Skin-Cancer Chemoprevention. N Engl J Med (2015) 373(17):1618–26. 10.1056/NEJMoa1506197 26488693

[25] George R Weightman W Russ GR Bannister KM Mathew TH. Acitretin for Chemoprevention of Non-melanoma Skin Cancers in Renal Transplant Recipients. Australas J Dermatol (2002) 43(4):269–73. 10.1046/j.1440-0960.2002.00613.x 12423433

[26] Massey PR Schmults CD Li SJ Arron ST Asgari MM Bouwes Bavinck JN Consensus-Based Recommendations on the Prevention of Squamous Cell Carcinoma in Solid Organ Transplant Recipients: A Delphi Consensus Statement. JAMA Dermatol (2021) 157:1219–26. 10.1001/jamadermatol.2021.3180 34468690 PMC9937447

[27] Stasko T Brown MD Carucci JA Euvrard S Johnson TM Sengelmann RD Guidelines for the Management of Squamous Cell Carcinoma in Organ Transplant Recipients. Dermatol Surg (2004) 30(4 2):642–50. 10.1111/j.1524-4725.2004.30150.x 15061849

[28] Lopez AT Carvajal RD Geskin L. Secondary Prevention Strategies for Nonmelanoma Skin Cancer. Oncology (Williston Park) (2018) 32(4):195–200. 29684233

[29] Jansen MHE Kessels J Nelemans PJ Kouloubis N Arits A van Pelt HPA Randomized Trial of Four Treatment Approaches for Actinic Keratosis. N Engl J Med (2019) 380(10):935–46. 10.1056/NEJMoa1811850 30855743

[30] Weinstock MA Thwin SS Siegel JA Marcolivio K Means AD Leader NF Chemoprevention of Basal and Squamous Cell Carcinoma with a Single Course of Fluorouracil, 5%, Cream: A Randomized Clinical Trial. JAMA Dermatol (2018) 154(2):167–74. 10.1001/jamadermatol.2017.3631 29299592 PMC5839275

[31] Kisling LA. Prevention Strategies. Orlando, FL: StatPearls (2022). 30725907

[32] Ulrich C Jurgensen JS Degen A Hackethal M Ulrich M Patel MJ Prevention of Non-melanoma Skin Cancer in Organ Transplant Patients by Regular Use of a Sunscreen: a 24 Months, Prospective, Case-Control Study. Br J Dermatol (2009) 161(3):78–84. 10.1111/j.1365-2133.2009.09453.x 19775361

[33] Werner RN Sammain A Erdmann R Hartmann V Stockfleth E Nast A The Natural History of Actinic Keratosis: a Systematic Review. Br J Dermatol (2013) 169(3):502–18. 10.1111/bjd.12420 23647091

[34] Tokez S Wakkee M Louwman M Noels E Nijsten T Hollestein L. Assessment of Cutaneous Squamous Cell Carcinoma (cSCC) *In Situ* Incidence and the Risk of Developing Invasive cSCC in Patients with Prior cSCC *In Situ* vs the General Population in the Netherlands, 1989-2017. JAMA Dermatol (2020) 156(9):973–81. 10.1001/jamadermatol.2020.1988 32609322 PMC7330830

[35] Nemer KM Council ML. Topical and Systemic Modalities for Chemoprevention of Nonmelanoma Skin Cancer. Dermatol Clin (2019) 37(3):287–95. 10.1016/j.det.2019.02.004 31084723

[36] Heppt MV Steeb T Niesert AC Zacher M Leiter U Garbe C Local Interventions for Actinic Keratosis in Organ Transplant Recipients: a Systematic Review. Br J Dermatol (2019) 180(1):43–50. 10.1111/bjd.17148 30188570

[37] Hasan ZU Ahmed I Matin RN Homer V Lear JT Ismail F Topical Treatment of Actinic Keratoses in Organ Transplant Recipients: a Feasibility Study for SPOT (Squamous Cell Carcinoma Prevention in Organ Transplant Recipients Using Topical Treatments). Br J Dermatol (2022) 187:324–37. 10.1111/bjd.20974 34988975 PMC9543168

[38] Harwood CA Leedham-Green M Leigh IM Proby CM. Low-dose Retinoids in the Prevention of Cutaneous Squamous Cell Carcinomas in Organ Transplant Recipients: a 16-year Retrospective Study. Arch Dermatol (2005) 141(4):456–64. 10.1001/archderm.141.4.456 15837863

[39] Giacalone S Spigariolo CB Bortoluzzi P Nazzaro G. Oral Nicotinamide: The Role in Skin Cancer Chemoprevention. Dermatol Ther (2021) 34(3):e14892. 10.1111/dth.14892 33595161

[40] Inman GJ Wang J Nagano A Alexandrov LB Purdie KJ Taylor RG The Genomic Landscape of Cutaneous SCC Reveals Drivers and a Novel Azathioprine Associated Mutational Signature. Nat Commun (2018) 9(1):3667. 10.1038/s41467-018-06027-1 30202019 PMC6131170

[41] Corchado-Cobos R García-Sancha N González-Sarmiento R Pérez-Losada J Cañueto J. Cutaneous Squamous Cell Carcinoma: From Biology to Therapy. Int J Mol Sci (2020) 21(8):2956. 10.3390/ijms21082956 32331425 PMC7216042

[42] Jiyad Z Olsen CM Burke MT Isbel NM Green AC. Azathioprine and Risk of Skin Cancer in Organ Transplant Recipients: Systematic Review and Meta-Analysis. Am J Transpl (2016) 16(12):3490–503. 10.1111/ajt.13863 27163483

[43] Ming M Zhao B Qiang L He Y-Y. Effect of Immunosuppressants Tacrolimus and Mycophenolate Mofetil on the Keratinocyte UVB Response. Photochem Photobiol (2015) 91(1):242–7. 10.1111/php.12318 25039758 PMC4294972

[44] Mittal A Colegio OR. Skin Cancers in Organ Transplant Recipients. Am J Transpl (2017) 17(10):2509–30. 10.1111/ajt.14382 28556451

[45] Gibson JAG Cordaro A Dobbs TD Griffiths R Akbari A Whitaker S The Association between Immunosuppression and Skin Cancer in Solid Organ Transplant Recipients: a Control-Matched Cohort Study of 2, 852 Patients. Eur J Dermatol (2021) 31(6):712–21. 10.1684/ejd.2021.4108 34427560

[46] Vos M Plasmeijer E van Bemmel B van der Bij W Klaver N Erasmus M Azathioprine to Mycophenolate Mofetil Transition and Risk of Squamous Cell Carcinoma after Lung Transplantation. J Heart Lung Transpl (2018) 37(7):853–9. 10.1016/j.healun.2018.03.012 29680587

[47] Wang M Mittal A Colegio OR. Belatacept Reduces Skin Cancer Risk in Kidney Transplant Recipients. J Am Acad Dermatol (2020) 82(4):996–8. 10.1016/j.jaad.2019.09.070 31589945

[48] Euvrard S Morelon E Rostaing L Goffin E Brocard A Tromme I Sirolimus and Secondary Skin-Cancer Prevention in Kidney Transplantation. N Engl J Med (2012) 367(4):329–39. 10.1056/NEJMoa1204166 22830463

[49] Hoogendijk-van den Akker JM Harden PN Hoitsma AJ Proby CM Wolterbeek R Bouwes Bavinck JN Two-year Randomized Controlled Prospective Trial Converting Treatment of Stable Renal Transplant Recipients with Cutaneous Invasive Squamous Cell Carcinomas to Sirolimus. J Clin Oncol (2013) 31(10):1317–23. 10.1200/JCO.2012.45.6376 23358973

[50] Dantal J Morelon E Rostaing L Goffin E Brocard A Tromme I Sirolimus for Secondary Prevention of Skin Cancer in Kidney Transplant Recipients: 5-Year Results. J Clin Oncol (2018) 36(25):2612–20. 10.1200/JCO.2017.76.6691 30016177

[51] Knoll GA Kokolo MB Mallick R Beck A Buenaventura CD Ducharme R Effect of Sirolimus on Malignancy and Survival after Kidney Transplantation: Systematic Review and Meta-Analysis of Individual Patient Data. BMJ : Br Med J (2014) 349:g6679. 10.1136/bmj.g6679 25422259 PMC4241732

[52] Berger SP Sommerer C Witzke O Tedesco H Chadban S Mulgaonkar S Two-year Outcomes in De Novo Renal Transplant Recipients Receiving Everolimus-Facilitated Calcineurin Inhibitor Reduction Regimen from the TRANSFORM Study. Am J Transpl (2019) 19(11):3018–34. 10.1111/ajt.15480 31152476

[53] Preterre J Visentin J Saint Cricq M Kaminski H Del Bello A Prezelin-Reydit M Comparison of Two Strategies Based on Mammalian Target of Rapamycin Inhibitors in Secondary Prevention of Non-melanoma Skin Cancer after Kidney Transplantation, a Pilot Study. Clin Transpl (2021) 35(3):e14207. 10.1111/ctr.14207 33369772

[54] Lim WH Russ GR Wong G Pilmore H Kanellis J Chadban SJ. The Risk of Cancer in Kidney Transplant Recipients May Be Reduced in Those Maintained on Everolimus and Reduced Cyclosporine. Kidney Int (2017) 91(4):954–63. 10.1016/j.kint.2016.11.008 28109543

[55] Bestard O Thaunat O Bellini MI Bohmig GA Budde K Claas F Alloimmune Risk Stratification for Kidney Transplant Rejection. Transpl Int (2022) 35:10138. 10.3389/ti.2022.10138 35669972 PMC9163827

[56] Otley CC Maragh SLH. Reduction of Immunosuppression for Transplant-Associated Skin Cancer: Rationale and Evidence of Efficacy. Dermatol Surg (2006) 31(2):163–8. 10.1111/j.1524-4725.2005.31038 15762208

[57] Dantal J Hourmant M Cantarovich D Giral M Blancho G Dreno B Effect of Long-Term Immunosuppression in Kidney-Graft Recipients on Cancer Incidence: Randomised Comparison of Two Cyclosporin Regimens. Lancet (1998) 351(9103):623–8. 10.1016/S0140-6736(97)08496-1 9500317

[58] Van Leeuwen MT Webster AC McCredie MRE Stewart JH McDonald SP Amin J Effect of Reduced Immunosuppression after Kidney Transplant Failure on Risk of Cancer: Population Based Retrospective Cohort Study. BMJ (2010) 340(11 2):c570–c. 10.1136/bmj.c570 20150194 PMC2820609

[59] Barozzi P Bonini C Potenza L Masetti M Cappelli G Gruarin P Changes in the Immune Responses against Human Herpesvirus-8 in the Disease Course of Posttransplant Kaposi Sarcoma. Transplantation (2008) 86(5):738–44. 10.1097/TP.0b013e318184112c 18791457

[60] Hall EC Engels EA Pfeiffer RM Segev DL. Association of Antibody Induction Immunosuppression with Cancer after Kidney Transplantation. Transplantation (2015) 99(5):1051–7. 10.1097/TP.0000000000000449 25340595 PMC4405385

[61] De Pasquale C Veroux M Indelicato L Sinagra N Giaquinta A Fornaro M Psychopathological Aspects of Kidney Transplantation: Efficacy of a Multidisciplinary Team. World J Transpl (2014) 4(4):267–75. 10.5500/wjt.v4.i4.267 PMC4274596 25540735

[62] Howell M Tong A Wong G Craig JC Howard K. Important Outcomes for Kidney Transplant Recipients: a Nominal Group and Qualitative Study. Am J Kidney Dis (2012) 60(2):186–96. 10.1053/j.ajkd.2012.02.339 22578839

[63] Howell M Wong G Rose J Tong A Craig JC Howard K. Patient Preferences for Outcomes after Kidney Transplantation: A Best-Worst Scaling Survey. Transplantation (2017) 101(11):2765–73. 10.1097/TP.0000000000001793 29064956

[64] Nickerson PW. What Have We Learned about How to Prevent and Treat Antibody‐mediated Rejection in Kidney Transplantation? Am J Transpl (2020) 20(S4):12–22. 10.1111/ajt.15859 32538535

[65] Borden ES Kang P Natri HM Phung TN Wilson MA Buetow KH Neoantigen Fitness Model Predicts Lower Immune Recognition of Cutaneous Squamous Cell Carcinomas Than Actinic Keratoses. Front Immunol (2019) 10:2799. 10.3389/fimmu.2019.02799 31849976 PMC6896054

[66] Stapleton CP Chang BL Keating BJ Conlon PJ Cavalleri GL. Polygenic Risk Score of Non‐melanoma Skin Cancer Predicts post‐transplant Skin Cancer across Multiple Organ Types. Clin Transpl (2020) 34(8):e13904. 10.1111/ctr.13904 32400091

[67] Seviiri M Law MH Ong JS Gharahkhani P Nyholt DR Hopkins P Polygenic Risk Scores Stratify Keratinocyte Cancer Risk Among Solid Organ Transplant Recipients with Chronic Immunosuppression in a High Ultraviolet Radiation Environment. J Invest Dermatol (2021) 141(12):2866–75.e2. 10.1016/j.jid.2021.03.034 34089721

[68] Chahal HS Lin Y Ransohoff KJ Hinds DA Wu W Dai HJ Genome-wide Association Study Identifies Novel Susceptibility Loci for Cutaneous Squamous Cell Carcinoma. Nat Commun (2016) 7:12048. 10.1038/ncomms12048 27424798 PMC4960294

[69] Siiskonen SJ Zhang M Li WQ Liang L Kraft P Nijsten T A Genome-wide Association Study of Cutaneous Squamous Cell Carcinoma Among European Descendants. Cancer Epidemiol Biomarkers Prev (2016) 25(4):714–20. 10.1158/1055-9965.EPI-15-1070 26908436 PMC4873347

[70] Sarin KY Lin Y Daneshjou R Ziyatdinov A Thorleifsson G Rubin A Genome-wide Meta-Analysis Identifies Eight New Susceptibility Loci for Cutaneous Squamous Cell Carcinoma. Nat Commun (2020) 11(1):820. 10.1038/s41467-020-14594-5 32041948 PMC7010741

[71] Asgari MM Wang W Ioannidis NM Itnyre J Hoffmann T Jorgenson E Identification of Susceptibility Loci for Cutaneous Squamous Cell Carcinoma. J Invest Dermatol (2016) 136(5):930–7. 10.1016/j.jid.2016.01.013 26829030 PMC4842155

[72] Geusau A Borik-Heil L Skalicky S Mildner M Grillari J Hackl M Dysregulation of Tissue and Serum microRNAs in Organ Transplant Recipients with Cutaneous Squamous Cell Carcinomas. Health Sci Rep (2020) 3(4):e205. 10.1002/hsr2.205 33251338 PMC7676459

[73] Blue ED Freeman SC Lobl MB Clarey DD Fredrick RL Wysong A Cutaneous Squamous Cell Carcinoma Arising in Immunosuppressed Patients: A Systematic Review of Tumor Profiling Studies. JID Innov (2022) 2(4):100126. 10.1016/j.xjidi.2022.100126 35620703 PMC9127418

[74] Wysong A Newman JG Covington KR Kurley SJ Ibrahim SF Farberg AS Validation of a 40-gene Expression Profile Test to Predict Metastatic Risk in Localized High-Risk Cutaneous Squamous Cell Carcinoma. J Am Acad Dermatol (2021) 84(2):361–9. 10.1016/j.jaad.2020.04.088 32344066

[75] Bottomley MJ Harden PN Wood KJ. CD8+ Immunosenescence Predicts Post-Transplant Cutaneous Squamous Cell Carcinoma in High-Risk Patients. J Am Soc Nephrol (2016) 27(5):1505–15. 10.1681/ASN.2015030250 26563386 PMC4849821

[76] Carroll RP Segundo DS Hollowood K Marafioti T Clark TG Harden PN Immune Phenotype Predicts Risk for Posttransplantation Squamous Cell Carcinoma. J Am Soc Nephrol (2010) 21(4):713–22. 10.1681/ASN.2009060669 20110382 PMC2844307

[77] Hope CM Grace BS Pilkington KR Coates PT Bergmann IP Carroll RP. The Immune Phenotype May Relate to Cancer Development in Kidney Transplant Recipients. Kidney Int (2014) 86(1):175–83. 10.1038/ki.2013.538 24429406

[78] Normand S-LT. The RECOVERY Platform. N Engl J Med (2021) 384(8):757–8. 10.1056/NEJMe2025674 32706531 PMC7386419

